# Chemistry and Bioactivity of Marine-Derived Bisabolane Sesquiterpenoids: A Review

**DOI:** 10.3389/fchem.2022.881767

**Published:** 2022-04-07

**Authors:** Cheng-Shou Li, Li-Ting Liu, Lei Yang, Jing Li, Xin Dong

**Affiliations:** Qingdao Hospital of Traditional Chinese Medicine (Qingdao Hiser Hospital), Qingdao, China

**Keywords:** marine-derived bisabolanes, sesquiterpenoids, chemical diversity, biological activities, lead compounds

## Abstract

Natural products, characterized by intriguing scaffold diversity and structural complexity, as well as significant agricultural and medicinal activities, have been a valuable source of agrochemicals/drugs development and have historically made a huge contribution to pharmacotherapy. Structurally, bisabolanes are a family of naturally occurring sesquiterpenoids that featured a hexatomic ring core incorporating with eight continuous carbons, which cause high structural variability along the alkyl side chain to form abundant functionalities. Moreover, apart from their interesting structures, bisabolanes have shown multitudinous bioactivities. Bisabolanes are distributed in a variety of marine invertebrates, terrestrial plant, and microbial sources. Interestingly, bisabolanes characterized from marine environment possess unique characteristics both structurally and biologically. A total of 296 newly-discovered bisabolanes were searched. Among them, 94 members were isolated from marine organisms. This review particularly focuses on the new bisabolanes characterized from marine organisms (covering from 2000 to 2021), including marine-derived fungi, algae, soft corals, and sponges, with emphasis on the diversity of their chemical structures as well as the novelty and differences between terrestrial and marine sources. Moreover, a wide range of bioactivities of marine-derived bisabolanes, including antimicrobial, anti-inflammatory, enzyme inhibitory, and cytotoxic properties, are presented herein, which is considered to be a promising resource for the discovery of new drug leads and agrochemicals.

## Introduction

Natural products (NPs) are secondary metabolites produced by plants, microorganisms, and animals through diversified biosynthetic pathways. NPs possess attractive structural diversity and complexity along with prominent biological properties, and hence have played an essential role in the discovery of lead compounds for new medicines (Butler, 2005). Historically, NPs and their derivatives have made a significant contribution to pharmacotherapy. The discovery of penicillin, the first broad-spectrum antibiotic, is a representative example, which, not only accelerated the course of pharmaceutical research but also saved thousands of lives (Zhang et al., 2020). NPs have been an inexhaustible reservoir for new drug development, which prove to be the cornerstone of modern pharmaceutical industry. It is estimated that approximately 60% of approved small-molecule based drugs between 1981 and 2019 are derived from NPs, indicating that there is still an urgent need to excavate more NPs with novel structures and promising bioactivities (Newman and Cragg, 2020).

Sesquiterpenoids represent structurally diverse NPs with extensive pharmacological activities, with more than 300 skeletons being reported up to now (Fraga, 2011). Bisabolanes are a family of sesquiterpenoids widely distributed in terrestrial plants, microorganisms, and marine organisms (algae, soft corals, and sponges) (Chen et al., 2021). Structurally, bisabolanes contain a hexatomic ring core that generally, but not always, features a 15-carbon skeleton. Bisabolanes appear to be among the simplest of all sesquiterpenoids, as most of them are monocyclic. However, they represent high complexity of cyclic sesquiterpenoids. These compounds incorporate an eight continuous carbons side chain, which cause high structural diversity to generate abundant functionalities including double bond, lactone, furan, pyran, and so on, by oxidation, reduction, esterification, and cyclization (Niu et al., 2020; Chen et al., 2021). Moreover, some unique members, such as norbisabolanes (Jian Li et al., 2015; Ge et al., 2021), dimers (Sun et al., 2012; Georgantea et al., 2013), glycosides (Lv et al., 2014), adducts (Xiao-Dong Li et al., 2015), have also been characterized. Chemically speaking, the findings of these fascinating molecules provide considerable chemical foundation in the course of new drugs and agricultural chemicals discovery. In addition, bisabolanes have been reported to possess extensive pharmacological activities, including but not limited to anti-inflammatory, insecticidal, antitumor, antimicrobial, and antiviral activities.

Marine environment has distinct ecological characteristics compared to terrestrial environment, such as high salinity, high pressure, extreme temperature, and low oxygen. To overcome those multiple extreme environmental stresses, marine organisms have evolved unique metabolic abilities to produce novel bioactive natural products. Marine natural products, therefore, possess unimaginable structural diversity and have brought immensely attention. A large amount of marine natural products have been discovered and reported, including polyketides, terpenoids, steroids, peptides, and nitrogen-containing compounds (Carroll et al., 2021). Compared to NPs isolated from terrestrial environment, marine natural products are often considered to be more diversiform and variable both structurally and biologically. For example, marine-derived heterocyclic alkaloids are challenging NPs possessing structurally unique skeletons arising from distinct amino acids (Zhang et al., 2020). Moreover, many of marine natural products are characterized as halogenated compounds, which are relatively few in terrestrial natural products. As for above-mentioned bisabolanes, it is believed that marine-derived bisabolanes may also have certain novelty and differences in contrast to terrestrial ones on structures and bioactivities. Therefore, to fully demonstrate the chemistry and bioactivity of newly-discovered marine-derived bisabolanes in recent years, we performed an in-depth investigation of these compounds, not only from marine sources but also from terrestrial sources. As a result, a total of 296 new bisabolanes and 93 references were searched, with 94 members isolated from marine organisms. They include 64 compounds (**1**–**64**) characterized from marine fungi, 6 compounds (**65**–**70**) from marine algae, 9 compounds (**71**–**79**) from soft corals, 15 compounds (**80**–**94**) from marine sponges. Moreover, another 27 compounds (**95**–**122**) from terrestrial fungi, 169 compounds (**123**–**291**) from terrestrial plants, and 5 compounds (**292**–**296**) from others were presented in Supplemental Materials (Supplementary Figures S1–S3). Pharmacological studies indicated the biological potential of these compounds, exhibiting antimicrobial, anti-inflammatory, enzyme inhibitory, cytotoxic, antimicroalgal, and antifouling properties. It should be pointed out that a review regarding to the chemistry and bioactivity of bisabolanes has been published very recently (Shu et al., 2021). The previous review by Shu et al. described 356 bisabolanes in total isolated from 24 families, primarily from Compositae, Zingiberaceae, Aspergillaceae, Halichondriidae, and Aplysiidae (Shu et al., 2021). Since the authors mixed up the marine and terrestrial bisabolanes for discussion, this review gave little information on marine bisabolanes, especially their structural features. In this study, this is the first attempt to summarize the chemical diversity of marine-derived bisabolanes, highlight their novelty and differences between terrestrial and marine sources, and provide a future perspective of their potential applications as lead compounds.

## Chemical Diversity of the Newly-Discovered Marine-Derived Bisabolanes

### Bisabolanes Characterized From Marine-Derived Fungi

**FIGURE 1:**
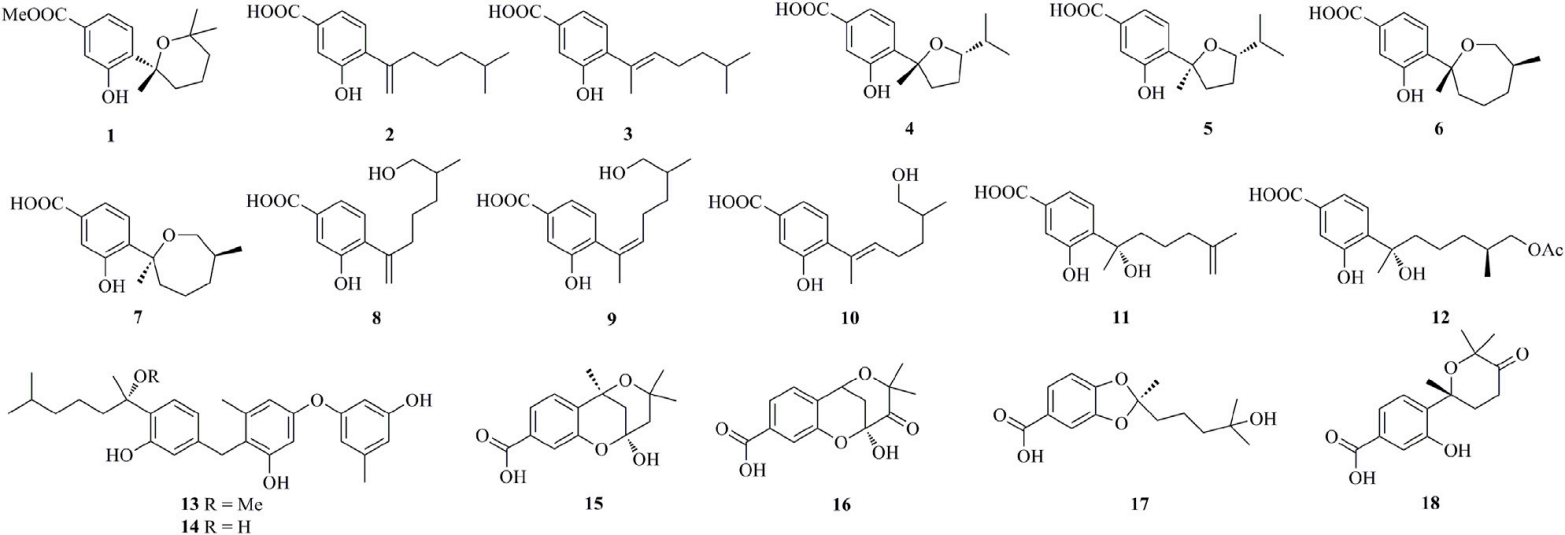
Bisabolanes characterized from marine-derived fungi (**1**–**18**).

**FIGURE 2:**
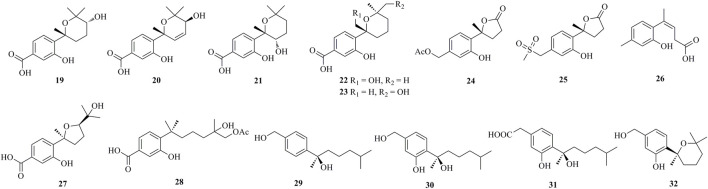
Bisabolanes characterized from marine-derived fungi (Continued) (**19**–**32**).

**FIGURE 3:**
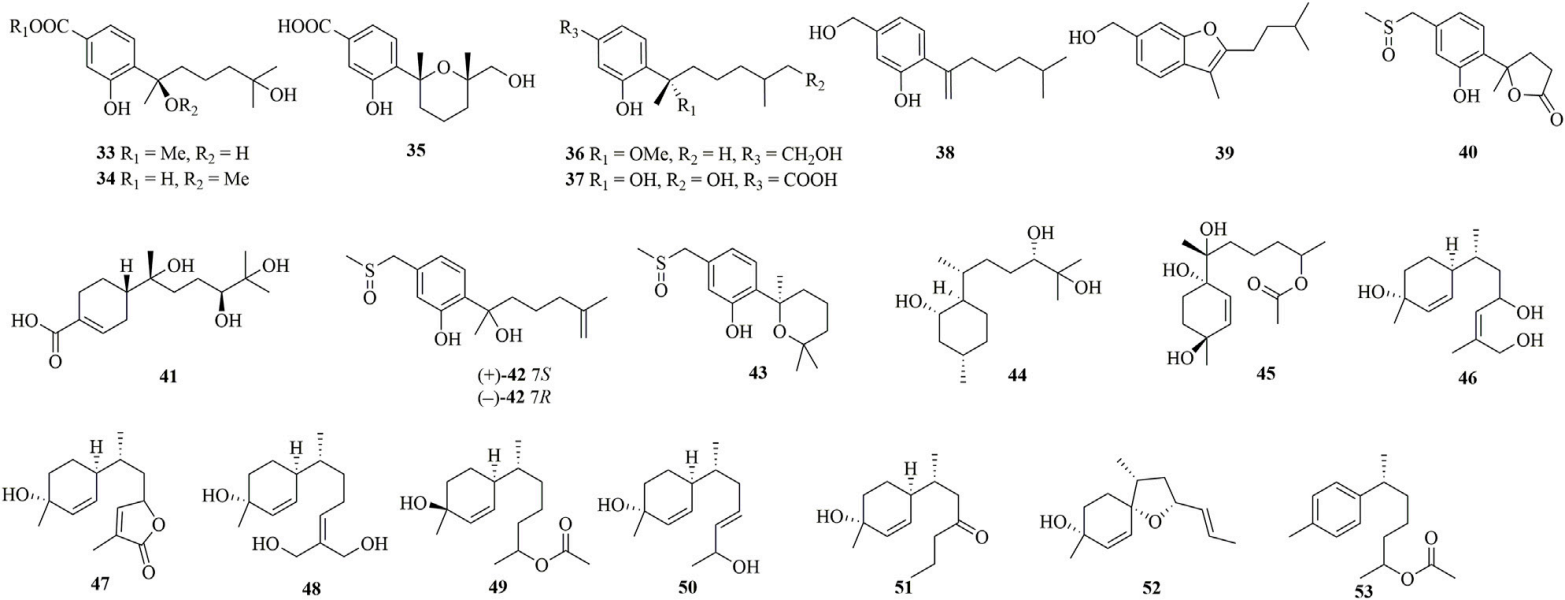
Bisabolanes characterized from marine-derived fungi (Continued) (**33**–**53**).

**FIGURE 4:**
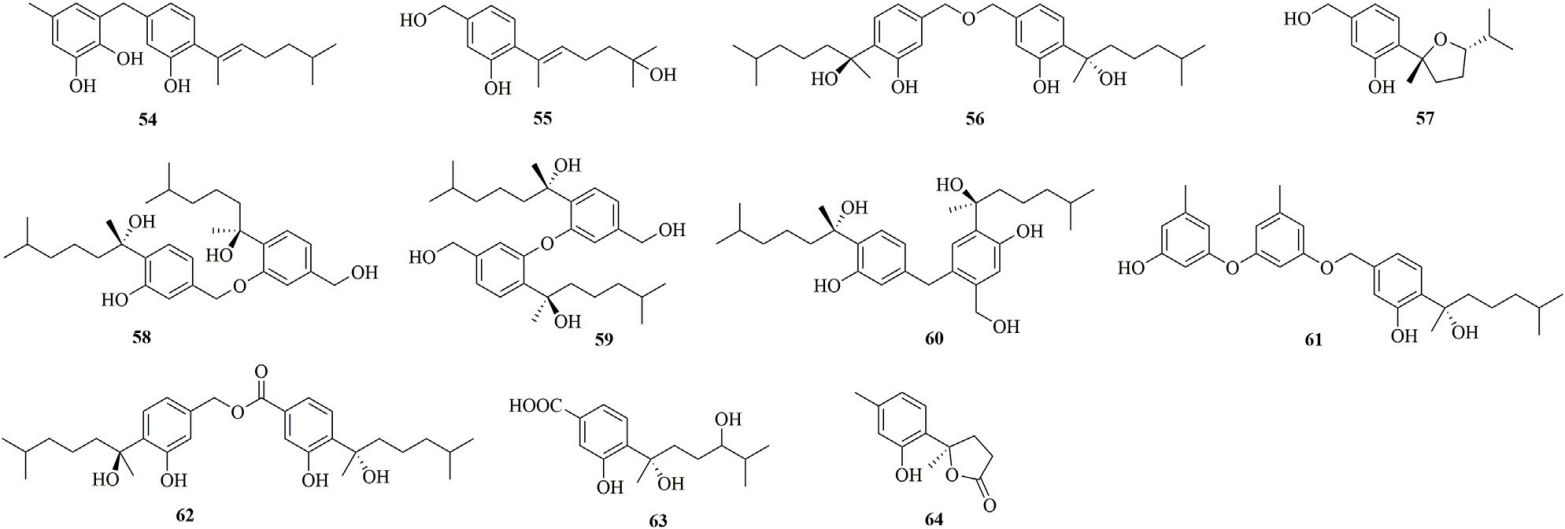
Bisabolanes characterized from marine-derived fungi (Continued) (**54**–**64**).

As shown in Figure 1, Figure 2, Figure 3, Figure 4, a total of 64 new bisabolanes (**1**–**64**) were characterized from marine-derived filamentous fungi. Bioassay-guided isolation of a marine-derived fungus *Aspergillus* sp. obtained from a gorgonian coral yielded three new phenolic bisabolanes, (+)-methyl sydowate (**1**), 7-deoxy-7,14-didehydrosydonic acid (**2**), and 7-deoxy-7,8-didehydrosydonic acid **3**) (Wei et al., 2010). **1** possessed a 2,2,6-trimethyltetrahydro-2*H*-pyran moiety, which was proved as a natural product, rather than an artifact during the isolation process. Chemical investigation of the mangrove-derived endophytic fungus *Aspergillus* sp. xy02 resulted in the isolation of seven new phenolic bisabolanes, namely (7*R*,10*S*)-7,10-epoxysydonic acid (**4**), (7*S*,10*S*)-7,10-epoxysydonic acid (**5**), (7*R*,11*S*)-7,12-epoxysydonic acid (**6**), (7*S*,11*S*)-7,12-epoxysydonic acid (**7**), 7-deoxy-7,14-didehydro-12-hydroxysydonic acid (**8**), (*Z*)-7-deoxy-7,8-didehydro-12-hydroxysydonic acid (**9**), and (*E*)-7-deoxy-7,8-didehydro-12-hydroxysydonic acid (**10**) (Wang et al., 2018). Compounds **4** and **5** were elucidated as a pair of isomers of the 7,10-epoxide of sydonic acid, while compounds **6** and **7** were found to form a 7,12-epoxide. Four new phenolic bisabolanes, (*S*)-(+)-11-dehydrosydonic acid **11**) and (7*S*,11*S*)-(+)-12-acetoxysydonic acid (**12**), and expansols A **13**) and B (**14**), were produced by the marine fungus *Penicillium expansum* 091,006, which was endogenous with the mangrove plant *Excoecaria agallocha* (Lu et al., 2010). Compounds **13** and **14** were the first examples of adducts containing both phenolic bisabolane sesquiterpenoid and diphenyl ether units.

Fourteen undescribed phenolic bisabolanes with diverse structures, asperbisabolanes A−N (**15**–**28**), were isolated from the deep sea sediment-derived fungus *A. sydowii* MCCC 3A00324 (Niu et al., 2020). **15** and **16** represented the first bisabolanes featuring a 6/6/6 tricyclic skeleton. **17** was characterized as a novel *seco*-bisabolane containing a rare dioxolane moiety, and **25** possessed a rare methylsulfonyl group. Four new bisabolanes, including aspergiterpenoid A (**29**), (−)-sydonol (**30**), (−)-sydonic acid (**31**), and (−)-5-(hydroxymethyl)-2-(2′,6′,6′-trimethyltetrahydro-2*H*-pyran-2-yl)phenol (**32**), were characterized from a marine sponge-sourced fungus *Aspergillus* sp. (Li et al., 2012). Two new phenolic bisabolanes, *ent*-aspergoterpenin C **33**) and 7-*O*-methylhydroxysydonic acid (**34**), were obtained from the culture extracts of *A. versicolor* SD-330, a deep-sea sediment-sourced fungus (Li et al., 2019a). **33** and **34** were considered as natural products through HPLC analysis, rather than artifacts produced by esterification or methylation. Then from the same fungus, another new compound 12-hydroxysydowic acid **35**) was isolated (Li et al., 2019b). Three new bisabolanes, (7*S*)-(+)-7-*O*-methylsydonol (**36**), (7*S*,11*S*)-(+)-12-hydroxysydonic acid (**37**), and 7-deoxy-7,14-didehydrosydonol (**38**), were isolated from the fungus *A. sydowii* by the addition of a DNA methyltransferase inhibitor 5-azacytidine (Chung et al., 2013).

Through chemical epigenetic manipulation strategy, the gorgonian-derived fungus *A. versicolor* XS-20090066 was found to produce a new bisabolane aspergillusene E (**39**) with a benzofuran moiety, by the addition of histone deacetylase inhibitor, suberoylanilide hydroxamic acid, and DNA methyltransferase inhibitor, 5-azacytidine (Wu et al., 2020). A pair of new S-containing norbisabolane enantiomers methylsulfinyl-1-hydroxyboivinianin A [(±)-**40**] were produced by the marine algal-derived endophytic fungus *P. chrysogenum* LD-201810 (Ge et al., 2021). **40** was proven to possess a rare methylsulfinyl substituent, and finally afforded individual enantiomers (+)-**40** and (−)-**40** by chiral HPLC. A new polychiral bisabolane with a 10,11-diol moiety named bisabolanoic acid A **41**) was isolated from the mangrove-derived endophytic fungus *Colletotrichum* sp. SCSIO KcB3-2 (Li et al., 2021). A pair of new bisabolanes enantiomers (±)-flavilane A (**42**), as well as a new derivative flavilane B (**43**), were isolated from the seawater-derived fungus *A. flavipes* 297 (Chen et al., 2021). **42** and **43** represented uncommon cases of phenolic bisabolanes incorporating with a methylsulfinyl group. A new bisabolane bisabolan-1,10,11-triol **44**) and a new norbisabolane 12-nor-11-acetoxybisabolen-3,6,7-triol **45**) were isolated from a marine algal-derived endophytic fungus *T. asperellum* cf44-2 (Song et al., 2018). In a systemic phytochemical investigation on a marine red alga-epiphytic isolate of *T. asperellum* Y6−2, eight new bisabolanes, trichobisabolins A−H (**46**–**53**), were finally obtained (Shi et al., 2019). Compounds **49**–**53** were rare nor-sesquiterpenes.

Four new phenolic bisabolanes **54**–**57** were isolated from the culture of mangrove endophytic fungus *A. flavus* QQSG-3 (Wu et al., 2018). **54** was a phenol-bisabolane adduct linked *via* a methylene, while **56** was a dimeric analogue of sydonol. Three rare phenolic bisabolane dimers, disydonols A−C (**58**–**60**), were acquired by the extensive investigation on a marine-derived fungus *Aspergillus* sp. (Sun et al., 2012). **58**–**60** were the dehydration dimeric metabolites of (*S*)-(+)-sydonol. Four new phenolic bisabolanes, peniciaculins A **61**) and B (**62**), (7*S*)-(−)-10-hydroxysydonic acid (**63**), and 1-hydroxyboivinianin A (**64**), were isolated from a deep sea sediment-derived fungus *P. aculeatum* SD-321 (Jian Li et al., 2015). **61** was a diphenyl ether-bisabolane adduct *via* an ether bond, whereas **62** represented the dimeric bisabolane with two monomers linking through an ester bond.

### Bisabolanes Characterized From Marine Algae

**FIGURE 5:**
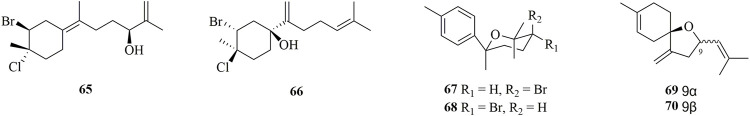
Bisabolanes characterized from marine algae (**65**–**70**).

Compounds **65**–**70** (Figure 5) were obtained from marine algae. Two new highly halogenated monocyclic bisabolanes, laurecomposins A **65**) and B (**66**), were discovered from a phytochemical investigation on the red alga *Laurencia composita* Yamada (Hu et al., 2020). Phytochemical studies on *L. okamurai*, a marine red alga, yielded four new bisabolane sesquiterpenes, okamurenes A−D (**67**–**70**) (Liang et al., 2012). **67** and **68** were brominated isomers, and **69** and **70** were dehalogenated 6,9-epoxybisabola-2,7 (14),10-triene. In the sense of chemotaxonomy, **67** and **68** represented the first members of brominated bisabolanes possessing a phenyl moiety among sesquiterpenes isolated from algae belonging to *Laurencia*.

### Bisabolanes Characterized From Soft Corals

**FIGURE 6:**
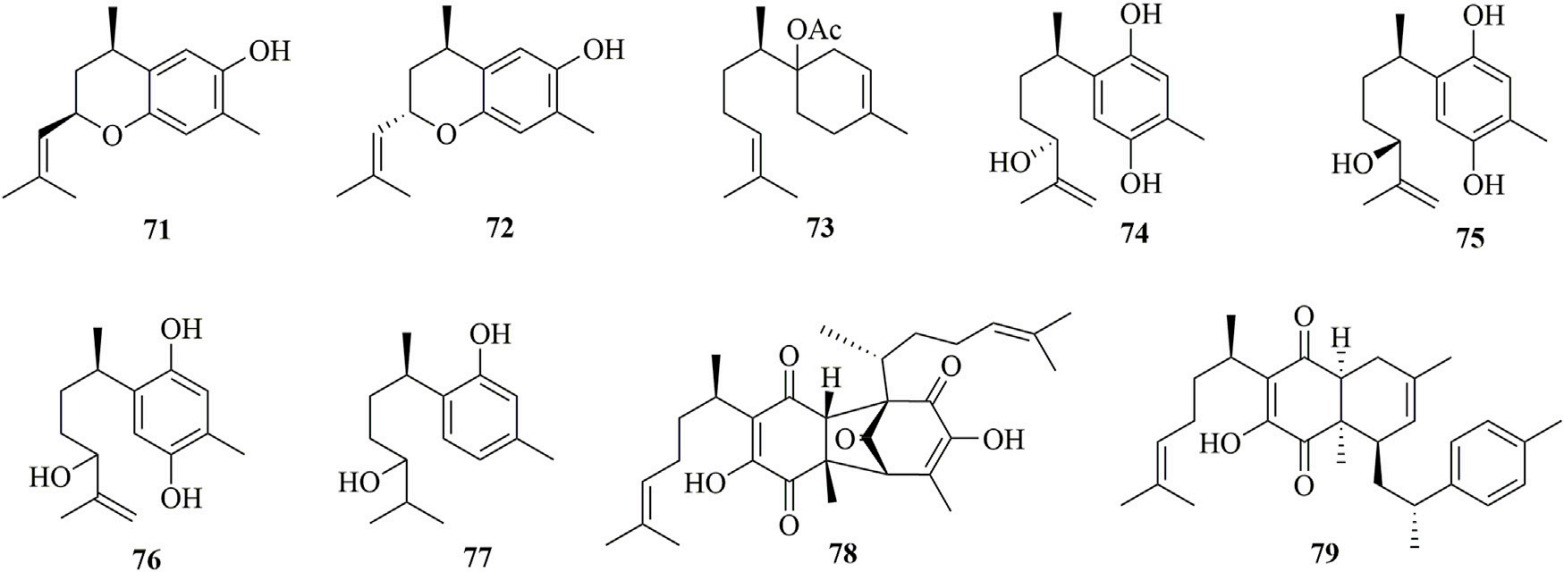
Bisabolanes characterized from soft corals (**71**–**79**).

Detailed chemical investigation of the tropical soft coral *Pseudopterogorgia rigida* led to the discovery of seven new bisabolanes **71**–**77** (Figure 6) (Georgantea et al., 2014). These obtained compounds belonged to phenolic bisabolanes, except for **73**, which was elucidated as acetyl-β-bisabolol. Moreover, **71** and **72** were epimers of 1,9-epoxy-4-hydroxy-α-curcumene. This species also produced minor constituents, perezoperezone **78**) and curcuperezone **79**) (Georgantea et al., 2013). **78** possessed a non-symmetrical dimeric structure through the formation of a five-membered ring, whereas **79** was a rare dimer synthesized by perezone and α-curcumene to form a tricyclic core.

### Bisabolanes Characterized From Marine Sponges

**FIGURE 7:**
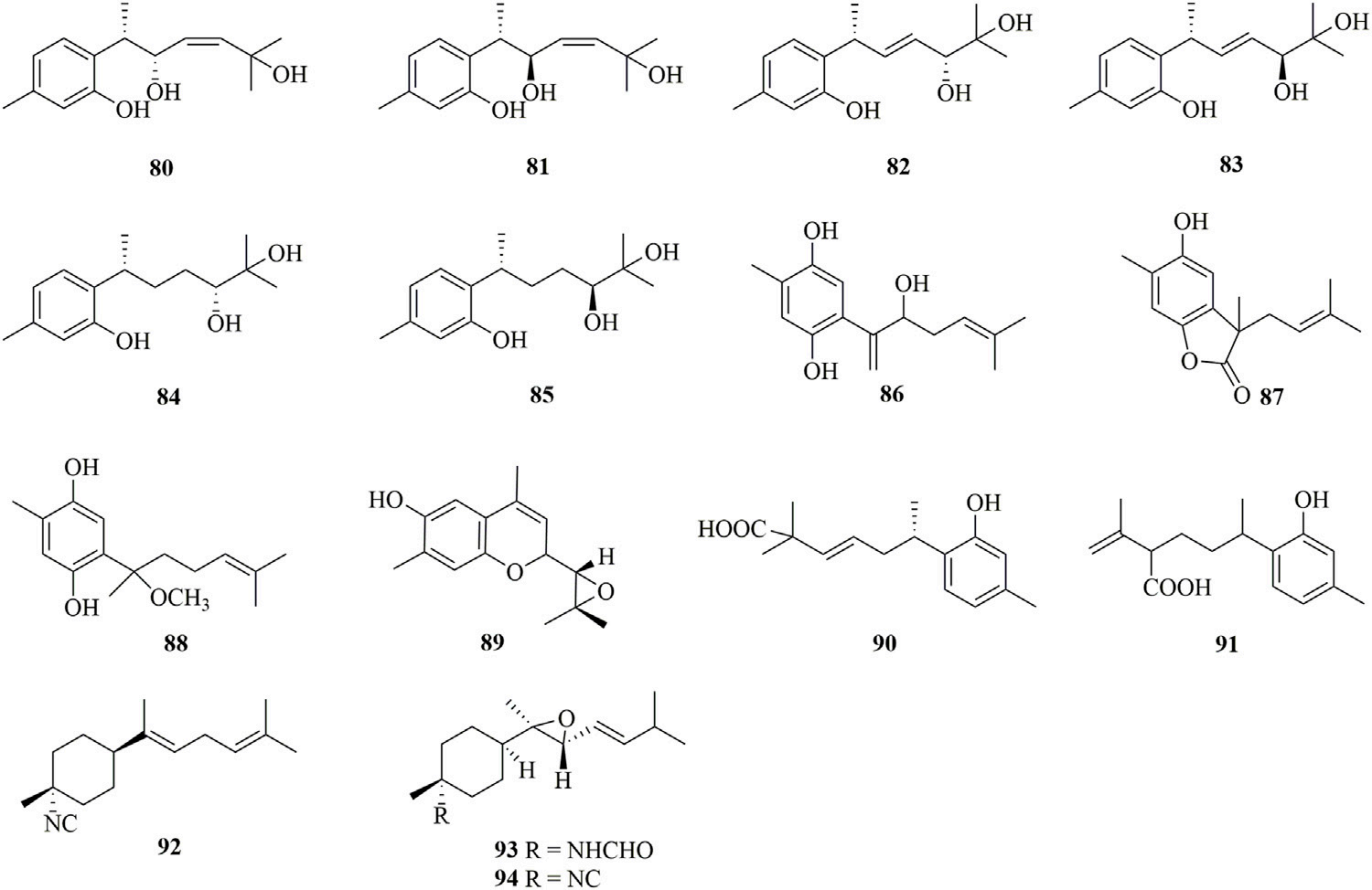
Bisabolanes characterized from marine sponges (**80**–**94**).

As shown in Figure 7, 15 bisabolanes were isolated and identified from marine sponges. Six new aromatic bisabolanes, plakordiols A−D (**80**–**83**), (7*R*, 10*R*)-hydroxycurcudiol (**84**), and (7*R*, 10*S*)-hydroxycurcudiol (**85**), were isolated from the marine sponge *Plakortis simplex* (Wang et al., 2021). Another four new aromatic bisabolanes, including 6-(3-hydroxy-6-methyl-1,5-heptadien-2-yl)-3-methylbenzene-1,4-diol (**86**), 4-hydroxy-3,7-dimethyl-7-(3-methylbut-2-en-1-yl)benzofuran-15-one (**87**), 6-(2-methoxy-5-methylhept-4-en-2-yl)-3-methylbenzene-1,4-diol (**88**), and 9-(3,3-dimethyloxiran-2-yl)-1,7-dimethyl-7-chromen-4-ol (**89**), were obtained from an organic extract of the marine sponge *Myrmekioderma* sp. (Costa et al., 2019). All of the isolated compounds represented cyclic bisabolanes bearing rich oxo functionality. Furthermore, chemical investigation of the Thai marine sponge *Myrmekioderma* sp. afforded two new aromatic bisabolanes, myrmekioperoxides A (**90**) and B **91**) (Wongbundit et al., 2020). A new monocyclic bisabolane (*E*)-3-isocyanobisabolane-7,10-diene **92**) containing an isonitrile group was isolated from the Okinawan sponge of the genus *Axinyssa* (Iwashima et al., 2002). Finally, two new rare epoxy-substituted nitrogenous bisabolanes, 3-formamido-7,8-epoxy-α-bisabolane **93**) and 3-isocyano-7,8-epoxy-α-bisabolane (**94**), were produced by the Hainan sponge *Axinyssa* sp. (Sun et al., 2010). **93** and **94** were α-bisabolanes with an 7,8-epoxy ring, which also contained a formamide functional group in **93** and an isonitrile group in **94**. It is worth mentioning that, although nitrogen-bearing bisabolanes are widely encountered in marine invertebrates, epoxy-substituted nitrogenous bisabolanes are rather rare.

## Biological Activities of Bisabolanes

### Antimicrobial Activity

Compound **1** exerted weak antibacterial efficacy against the Gram positive strain *Staphylococcus aureus* with an inhibition zone of 11 in diameter at the concentration of 100 μg/ml (Wei et al., 2010). Compounds **5**, **6**, **8**, and **10** showed mild antibacterial effect on *S. aureus* with the IC_50_ values of 32.2, 36.0, 41.9, and 31.5 μM, respectively (Wang et al., 2018). Compound **30** was active against the pathogenic bacteria *S. albus* and *Micrococcus tetragenus* with the MICs of 5.00 and 1.25 μM, while **32** on *S. albus* and *Bacillus subtilis* with the MICs of 5.00 and 2.50 μM, respectively (Li et al., 2012). Compounds **33**–**35** exhibited high antibacterial activity against zoonotic pathogenic bacteria *Escherichia coli*, *Edwardsiella tarda*, *Vibrio harveyi*, and *V. parahaemolyticus*, with MICs no higher than 8.0 μg/ml (Li et al., 2019a; Li et al., 2019b). **39** possessed activity against *S. epidermidis* and *S. aureus* with MICs of 8 and 16 μg/ml. Moreover, **39** also showed antifungal activity toward *Candida albicans* and *C. tropicalis* with MICs of 64 and 32 μg/ml, respectively (Wu et al., 2020). **44** and **45** showed weak activity against four marine pathogenic bacteria, *V. parahaemolyticus*, *V. anguillarum*, *V. harveyi*, and *V. splendidus*, with the inhibitory zone diameters of 6.3–7.5 mm at 20 μg/disk (Song et al., 2018). Compound **61** demonstrated inhibitory activity against aquatic pathogens *Micrococcus luteus* and *V. alginolyticus* with MICs of 1.0 and 2.0 μg/ml, respectively (Xiao-Dong Li et al., 2015). Moreover, the dimer **62** and norbisabolane **64** showed selective activity against *E. tarda* and *V. harveyi*, with MICs of 8.0 and 4.0 μg/ml. The halogenated bisabolanes **65** and **66** exhibited not only potent antifungal activity against *Microsporum gypseum*, with MICs of 4 and 8 μg/ml, but also considerable antibacterial activity against *S. aureus* with MICs of 26.8 and 15.4 μg/ml, respectively (Hu et al., 2020).

### Cytotoxic Activity

Compound **13**, a new adduct of phenolic bisabolane and diphenyl ether, possessed moderate cytotoxicity against HL-60 tumor cells with an IC_50_ value of 15.7 μM, and **14** inhibited higher activity against A549 and HL-60 cells with IC_50_ values of 1.9 and 5.4 μM, respectively (Lu et al., 2010). The condensation between phenolic bisabolanes and diphenyl ethers appear to enhance cytotoxicity significantly. The sulfurated compounds **42** and **43** exhibited moderate cytotoxicity against MKN-45 and HepG2 cells, with the IC_50_ values ranging from 19.8 to 30.1 μg/ml, which indicated that the methylsulfinyl group could strengthen cytotoxicity (Chen et al., 2021). The dimmers **58** and **60** showed pronounced cytotoxic activities against HepG-2 and Caski cell lines with IC_50_ values of 2.91–12.40 μg/ml, whereas **59** was relatively noncytotoxic (IC_50_ > 100 μg/ml) (Sun et al., 2012).

### Anti-Inflammatory Activity

Compounds **15**–**28** were evaluated inhibition effects against nitric oxide (NO) secretion in LPS-activated BV-2 microglia cells. **20** and **26** possessed the inhibition rates of 46.4 and 56.8% at a concentration of 10 μM. Structure–activity relationship analysis indicated that the double bond at Δ^7,8^ in bisabolane may strengthen the inhibition of NO secretion. Molecular mechanism study revealed that **26** inhibited the NF-κB-activated pathway in a dose-dependent manner (Niu et al., 2020).

### Enzyme Inhibitory Activity

The polychiral bisabolane **41** displayed moderate inhibitory activity against acetylcholinesterase (AChE) with an IC_50_ value of 2.2 μM. *In silico* molecular docking analysis indicated that **41** may bind to the active site residue TYR121 by hydrogen bonds (Li et al., 2021). **54** and **56** showed strong α-glucosidase inhibitory effects with IC_50_ values of 4.5 and 3.1 μM, respectively (Wu et al., 2018).

### Other Activities

Compound **32** not only showed selective antibacterial activity but also possessed significant antifouling activity. **32** drastically inhibited the larval settlement of the barnacle *Balanus amphitrite* at a concentration of 25.0 μg/ml (Li et al., 2012). **36** and **37** showed anti-diabetic activity, which increased medium glucose consumption of the differentiated 3T3-L1 adipocytes in comparison with the insulin treatment (Chung et al., 2013). **39** displayed antifouling activity against bryozoan larvae of *Bugula neritina* with the EC_50_ and LC_50_ values of 6.25 and 25 μg/ml, respectively (Wu et al., 2020). The norbisabolane **45** possessed antimicroalgal potency against the marine phytoplankton *Heterosigma akashiwo* with an IC_50_ value of 8.4 μg/ml (Song et al., 2018). Compounds **49**–**53** exhibited growth inhibition against four marine phytoplanktons (*H. akashiwo*, *Prorocentrum donghaiense*, *Chattonella marina*, and *Karlodinium veneficum*) with IC_50_s ranging from 2.1–85 μg/ml (Shi et al., 2019). It appeared that the acetoxy group at C-11 could significantly enhance inhibitory ability against marine phytoplanktons. Zebrafish Nile red fat metabolism assay revealed that compounds **86**, **87**, and **89** had significant lipid-reducing activity with IC_50_ values of 7.89, 12.61, and 1.22 μM, respectively (Costa et al., 2019). **92** containing an isonitrile group exerted strong brine shrimp lethal activity with an LC_50_ of 0.1 μg/ml (Iwashima et al., 2002).

## Discussions and Conclusion

### The Novelty of Bisabolanes Characterized From Fungi and Differences Between Terrestrial and Marine Sources

**FIGURE 8:**
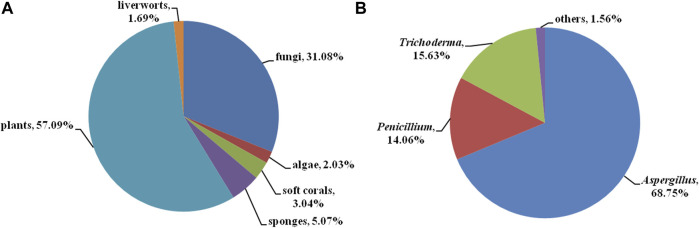
**(A)** Source categories of the described bisabolanes; **(B)** Categories of the marine fungi-derived bisabolanes.

Bisabolanes, a unique family of naturally occurring sesquiterpenoids, have been a valuable reservoir for searching for new lead compounds with medical and/or agricultural potential. These compounds are widely distributed in terrestrial plants, microorganisms, and marine organisms (algae, soft corals, and sponges) (Figure 8A). This review summarizes 296 newly-discovered bisabolanes in the past few years. Among them, 92 compounds, accounting for 31.08% of all described bisabolanes, were characterized from fungi [including marine-derived **1**–**64** (Figure 1, Figure 2, Figure 3, Figure 4) and terrestrial **95**–**122** (Supplementary Figure S1 in Supplemental Materials], especially filamentous fungi. The filamentous fungi *Aspergillus*, *Penicillium*, and *Trichoderma* are dominant genera as producers of these compounds, with 44, 9, and 10 members described, respectively (Figure 8B). Structurally, bisabolanes characterized from fungi are mainly phenolic bisabolanes, which featured a *para*-alkylated benzene ring framework comprising a side chain with an eight consecutive carbons. Their structure variability is due to the transformation of the side chain, such as oxidation, reduction, esterification, and cyclization, to generate abundant functionalities including lactone (eg. compounds **6**, **17**, **22**, **35**), double bond (e.g., compounds **8**–**10**), furan (e.g., compounds **4**, **5**, **39**), pyran (e.g., compounds **1**, **32**, **43**), and so on. Moreover, fungi also produce rare bisabolane dimers. Representative examples are compounds **13** and **14**, **58**–**62**, and **120**–**122**. It should be pointed out that, the endophytic fungi, an ecological group generally resided in plants and symbiotic with their hosts, are considered as an important producer of bisabolanes. Endophytic fungi possess a complicated interaction with their host plants, thus they have evolved novel metabolic pathways to produce the same or similar secondary metabolites as the hosts. Therefore, it is a more talented pathway to isolate bisabolanes from fungal endophytes, rather than the medicinal plants.

Compared to bisabolanes isolated from terrestrial fungi, some of bisabolanes from marine-derived fungi possess unique functionalities. For example, the sulfurated bisabolanes were exclusively isolated from marine fungi, including **25** with a rare methylsulfonyl group isolated from the deep sea sediment-derived fungus *A. sydowii* MCCC 3A00324 (Niu et al., 2020), **40** and **42**–**43** incorporating with a rare methylsulfinyl group isolated from the marine algal-derived endophytic fungus *P. chrysogenum* LD-201810 (Ge et al., 2021) and the seawater-derived fungus *A. flavipes* 297 (Chen et al., 2021), respectively. **52** isolated from a marine red alga-epiphytic isolate of *T. asperellum* Y6−2, represented a rare example of nor-sesquiterpenes with a spiro system (Shi et al., 2019). It is believed that marine-derived fungi suffered more survival stress than terrestrial fungi, and thus evolved more flexible metabolic pathways by producing novel secondary metabolites.

### Characterization of Terrestrial and Marine-Derived Bisabolanes

Apart from marine-derived fungi, a total of 30 compounds (**65**–**94**) were characterized from marine organisms, including marine plants algae and marine invertebrates, soft corals and sponges. It is well-known that marine organisms, which are suffered from high salinity, high pressure, extreme temperature, and low oxygen, possess unique metabolic pathways that are quite different from that of terrestrial organisms. Accordingly, marine-derived natural products tend to have unexpected structural types. For example, compounds **78** and **79**, two unprecedented non-symmetrical tricyclic dimers were characterized from the tropical soft coral *Pseudopterogorgia rigida* (Georgantea et al., 2013). In addition, some highly halogenated bisabolanes, such as **65**–**68**, were isolated from the marine algae belonging to *Laurencia* (Liang et al., 2012; Hu et al., 2020). With this consideration, it is believed that marine organisms, which have emerged as a valuable frontier for the discovery of new lead compounds, continue to be a rich source of structurally diverse bisabolanes.

Most of the described bisabolanes, apparently, are isolated from terrestrial plants. As shown in Figure 8A and Supplementary Figure S2, 169 compounds (**123**–**291**, accounting for 57.09% of all bisabolanes) are scattered across a variety of species belonging to the genera *Ligularia*, *Parasenecio*, *Curcuma*, and so on. The chemical types of plant-derived bisabolanes are relatively common. Most of them are featured as highly oxygenated monocyclic bisabolanes bearing a cyclohexane ring. Interestingly, some bisabolanes, such as **184**, **187**–**190**, **196**, and **201**, are uncommon chlorinated derivatives. It is generally considered that halogenated compounds can be exclusively isolated from the marine environment. The isolation of halogenated bisabolanes from terrestrial plants indicated that terrestrial plants may also possess great potentiality in finding novel halogenated compounds.

### Bioactivity Diversity and Potential Applications

**FIGURE 9:**
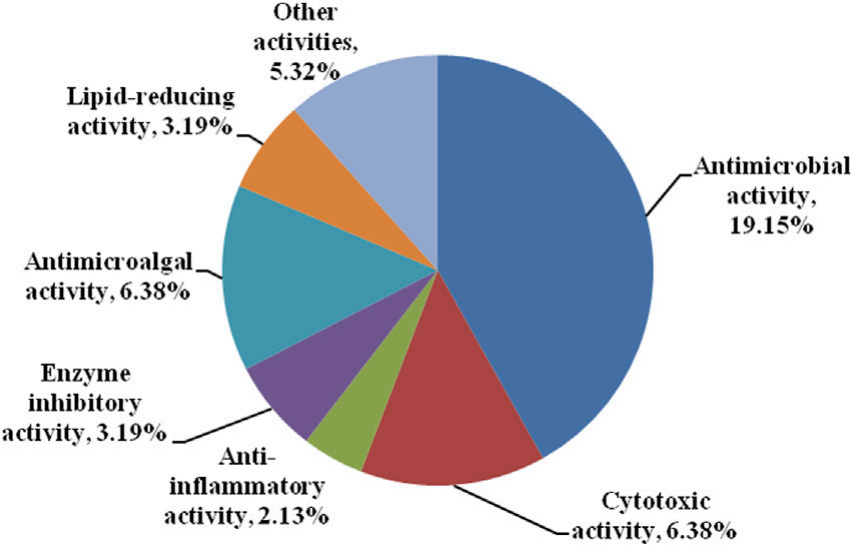
Bioactivity categories of the reported bisabolanes.

Bisabolanes are also attractive for their various and potent biological activities. As shown in Figure 9 and Table 1, the reported bisabolanes exhibited an extensive range of biological activities, including antimicrobial, anti-inflammatory, enzyme inhibitory, cytotoxic, antimicroalga, and other activities. Among the 94 described marine-derived bisabolanes, 43 compounds were found to possess moderate to potent biological activities. 18 compounds showed promising antibacterial or antifungal activity, while 6 compounds possessed various degrees of cytotoxic activity. Most importantly, some of them possessed significant bioactivities, which were higher than the positive controls. For example, **14** inhibited potent cytotoxic activity against A549 and HL-60 cells with IC_50_ values of 1.9 and 5.4 μM, respectively (Lu et al., 2010). **33**–**35** exhibited strong antibacterial activity against *E. coli*, *E. tarda*, *V. harveyi*, and *V. parahaemolyticus*, with MICs no higher than 8.0 μg/ml (Li et al., 2019a; Li et al., 2019b). In addition, bisabolanes characterized from terrestrial fungi and plants also possess promising activities. Compounds **118** and **119**, two dicyclic bisabolanes produced by the fungus *Pleurotus cystidiosus* were found to have significant cytotoxicity at the nM level. Especially, **119** exhibited the strongest effect on two human prostate cancer DU-145 and C42B cells, with the IC_50_s of 28 and 52 nM. Further pharmacological studies indicated that **119** induced the apoptosis of DU-145 cells (Zheng et al., 2015). The dicyclic **141** obtained from the rhizomes of *Curcuma longa* displayed more potent anti-inflammatory activity against the LPS-activated production of NO (IC_50_, 25.5 μM), when compared with the positive control dexamethasone (IC_50_, 33.6 μM) (Cheng et al., 2019). The aromatic bisabolanes **182** and **183** potently inhibited α-glucosidase with the IC_50_ values of 14.9 and 19.4 μM, respectively, approximately 30 times stronger than the positive control acarbose (IC_50_, 496 μM) (Song et al., 2021). The attractive bioactivities make many of these compounds suitable lead compounds or even candidates for the development of new medicines and agrochemicals and may trigger further pharmacological and synthesis studies.

**TABLE 1:** The environment source, producer, and biological activities of compounds **1**–**94**.

Compounds	Source	Producer	Biological activities	References
(+)-methyl sydowate (**1**), 7-deoxy-7,14-didehydrosydonic acid (**2**), and 7-deoxy-7,8-didehydrosydonic acid (**3**)	Marine fungi	*Aspergillus* sp.	Antibacterial activity	Wei et al. (2010)
(7*R*,10*S*)-7,10-epoxysydonic acid (**4**), (7*S*,10*S*)-7,10-epoxysydonic acid (**5**), (7*R*,11*S*)-7,12-epoxysydonic acid (**6**), (7*S*,11*S*)-7,12-epoxysydonic acid (**7**), 7-deoxy-7,14-didehydro-12-hydroxysydonic acid (**8**), (*Z*)-7-deoxy-7,8-didehydro-12-hydroxysydonic acid (**9**), and (*E*)-7-deoxy-7,8-didehydro-12-hydroxysydonic acid (**10**)	Marine fungi	*Aspergillus* sp. xy02	Antibacterial activity	Wang et al. (2018)
(*S*)-(+)-11-dehydrosydonic acid (**11**) and (7*S*,11*S*)-(+)-12-acetoxysydonic acid (**12**), and expansols A (**13**) and B (**14**)	Marine fungi	*Penicillium expansum*	Cytotoxic activity	Lu et al. (2010)
asperbisabolanes A−N (**15**–**28**)	Marine fungi	*A. sydowii* MCCC 3A00324	Anti-inflammatory activity	Niu et al. (2020)
aspergiterpenoid A (**29**), (−)-sydonol (**30**), (−)-sydonic acid (**31**), (−)-5-(hydroxymethyl)-2-(2′,6′,6′-trimethyltetrahydro-2*H*-pyran-2-yl)phenol (**32**)	Marine fungi	*Aspergillus* sp	Antibacterial and antifouling activity	Li et al. (2012)
*ent*-aspergoterpenin C (**33**), 7-*O*-methylhydroxysydonic acid (**34**)	Marine fungi	*A. versicolor* SD-330	Antibacterial activity	Li et al. (2019a)
12-hydroxysydowic acid (**35**)	Marine fungi	*A. versicolor* SD-330	Antibacterial activity	Li et al. (2019b)
(7*S*)-(+)-7-*O*-methylsydonol (**36**), (7*S*,11*S*)-(+)-12-hydroxysydonic acid (**37**), and 7-deoxy-7,14-didehydrosydonol (**38**)	Marine fungi	*A. sydowii*	Anti-diabetic and anti-inflammatory activity	Chung et al. (2013)
aspergillusene E (**39**)	Marine fungi	*A. versicolor* XS-20090066	Antimicrobial and antifouling activity	Wu et al. (2020)
methylsulfinyl-1-hydroxyboivinianin A (**40**)	Marine fungi	*P. chrysogenum* LD-201810	Antifungal activity	Ge et al. (2021)
bisabolanoic acid A (**41**)	Marine fungi	*Colletotrichum* sp. SCSIO KcB3-2	Enzyme inhibitory activity	Li et al. (2021)
(±)-flavilane A (**42**), flavilane B (**43**)	Marine fungi	*A. flavipes* 297	Cytotoxic activity	Chen et al. (2021)
bisabolan-1,10,11-triol (**44**), 12-nor-11-acetoxybisabolen-3,6,7-triol (**45**)	Marine fungi	*T. asperellum* cf44-2	Antibacterial and antimicroalgal activity	Song et al. (2018)
trichobisabolins A−H (**46**–**53**)	Marine fungi	*T. asperellum* Y6−2	Antimicroalgal activity	Shi et al. (2019)
**54**–**57** (names ungiven)	Marine fungi	*A. flavus* QQSG-3	Enzyme inhibitory activity	Wu et al. (2018)
disydonols A−C (**58**–**60**)	Marine fungi	*Aspergillus* sp	Cytotoxic activity	Sun et al. (2012)
peniciaculins A (**61**) and B (**62**), (7*S*)-(−)-10-hydroxysydonic acid (**63**), and 1-hydroxyboivinianin A (**64**)	Marine fungi	*P. aculeatum* SD-321	Antibacterial activity	Jian Li et al. (2015)
laurecomposins A (**65**) and B (**66**)	Algae	*Laurencia composita* Yamada	Antimicroalgal activity	Hu et al. (2020)
okamurenes A−D (**67**–**70**)	Algae	*L. okamurai*	No brine shrimp lethal activity	Liang et al. (2012)
**71**–**77** (names ungiven)	Soft corals	*Pseudopterogorgia rigida*	Unreported	Georgantea et al. (2014)
perezoperezone (**78**) and curcuperezone (**79**)	Soft corals	*Pseudopterogorgia rigida*	Unreported	Georgantea et al. (2013)
plakordiols A−D (**80**–**83**), (7*R*, 10*R*)-hydroxycurcudiol (**84**), and (7*R*, 10*S*)-hydroxycurcudiol (**85**)	Sponges	*Plakortis simplex*	No cytotoxic and antibacterial activity	Wang et al. (2021)
6-(3-hydroxy-6-methyl-1,5-heptadien-2-yl)-3-methylbenzene-1,4-diol (**86**), 4-hydroxy-3,7-dimethyl-7-(3-methylbut-2-en-1-yl)benzofuran-15-one (**87**), 6-(2-methoxy-5-methylhept-4-en-2-yl)-3-methylbenzene-1,4-diol (**88**), and 9-(3,3-dimethyloxiran-2-yl)-1,7-dimethyl-7-chromen-4-ol (**89**)	Sponges	*Myrmekioderma* sp	Lipid-reducing activity	Costa et al. (2019)
myrmekioperoxides A (**90**) and B (**91**)	Sponges	*Myrmekioderma* sp	Unreported	Wongbundit et al. (2020)
(*E*)-3-isocyanobisabolane-7,10-diene (**92**)	Sponges	*Axinyssa* sp	Brine shrimp lethal activity	Iwashima et al. (2002)
3-formamido-7,8-epoxy-α-bisabolane (**93**) and 3-isocyano-7,8-epoxy-α-bisabolane (**94**)	Sponges	*Axinyssa* sp	No cytotoxic activity	Sun et al. (2010)

In conclusion, bisabolane-type sesquiterpenoids, especially those isolated from marine organisms are considered to be nonnegligible natural products both structurally and biologically. This review systematically summarizes 296 newly reported bisabolanes characterized from fungi, marine algae, soft corals, marine sponges, terrestrial plants, and the others. Among them, 94 members were isolated from marine organisms. The following pharmacological studies indicated the biological potential of these compounds, which exhibited antimicrobial, anti-inflammatory, enzyme inhibitory, cytotoxic, antimicroalgal, and antifouling properties. This is the first attempt to focus on the chemistry and bioactivity of marine-derived bisabolanes, aiming to summarize the chemical diversity of marine-derived bisabolanes, highlight their novelty and differences between terrestrial and marine sources, and provide a future perspective of their potential application prospect as lead compounds. It is believed that in the near future, the research on these fascinating compounds will be increasingly abundant and conducive to the development of new drugs and agrochemicals.
