# Supplementary material for: Chemistry and Bioactivity of Marine-Derived Bisabolane Sesquiterpenoids: A Review
Source: Front Chem. 2022 Apr 7;10:881767. doi: 10.3389/fchem.2022.881767 (PMC9021493; doi:10.3389/fchem.2022.881767)
Supplement: Supplementary file 1 [file DataSheet1.docx]

*Supplementary Materials*

Chemistry and Bioactivity of Marine-Derived Bisabolane Sesquiterpenoids: A Review

Cheng-Shou Li^1^, Li-Ting Liu^1^, Lei Yang^1^, Jing Li^1*^, and Xin Dong^1*^

^1^ Qingdao Hospital of Traditional Chinese Medicine (Qingdao Hiser Hospital), Qingdao 266033, China

*** Correspondence:**Xin Dong
dongxin198901@163.com

Jing Li
lijing20220128@163.com

Keywords: marine-derived bisabolanes; sesquiterpenoids; chemical diversity; biological activities; lead compounds

## Bisabolanes characterized from terrestrial fungi

**Figure S1.** Bisabolanes characterized from terrestrial fungi (**95**–**122**).

A pair of new bisabolane enantiomers, phomoterpenes A and B [(±)-**95**] were isolated from *Phomopsis prunorum* F4-3, an endophytic fungus obtained from the leaves of *Hypericum ascyron* (Qu et al. 2020). As many reports indicated that several phenolic bisabolanes were produced naturally as racemic mixtures, the enantiomeric separation of (±)-**95** by chiral HPLC successfully afforded individual enantiomers (+)-**95** and (−)-**95**. Aspergoterpenin A (**96**), a new norbisabolane featuring a characteristic ketal bridged-ring moiety, along with three new bisabolane derivatives, aspergoterpenin B−D (**97**−**99**), was isolated from the endophytic fungus *A. versicolor* (Guo et al. 2018). Zopfiellins A−C (**100**−**102**), three undescribed bisabolanes were isolated from cultures of the endophytic fungus *Zopfiella* sp. (Sun et al. 2020). **100** and **101** contained an α,β-unsaturated cyclohexenone. In addition, **101** was elucidated as a rare *tri*-norbisabolane. Three novel bisabolanes (**103**−**105**, names ungiven) with a cyclohexanone core were obtained from the fungus *Penicillium* sp. (Stierle et al. 2004). Trichoderic acid (**106**), a new bisabolane containing the 1,8-epoxy bicyclic nucleus, was isolated from *Trichoderma* sp. PR-35, an endophyte of *Paeonia delavayi* (Wu et al. 2011). Chemical studies of the fungus *Antrodiella gypsea* led to the acquisition of a new bisabolane, gypseatriol (**107**) (Zhao et al. 2016). A new monocyclic bisabolane inonolane A (**108**) was isolated from the medicinal fungus *Inonotus vaninii* (Yang et al. 2013). **108** represented the first bisabolane from the genus *Inonotus*, indicating its chemotaxonomic significance. An investigation on chemical constituents of the fungus *I. rickii* yielded three new bisabolanes, inonotic acid A (**109**), 3-*O*-formyl inonotic acid A (**110**), and inonotic acid B (**111**) (Chen et al. 2014). Nidulal (**112**), a new bisabolane with a 1,4-disubstituted cyclohexane and a lactol ring, as well as niduloic acid (**113**) with a cyclohexene ring and a 2,4-disubstituted furan were isolated from the fungus belonging to basidiomycete *Nidula Candida* (Erkel et al. 1996). Virgineol (**114**), a highly oxygenated bisabolane possessing 6/5/5 tricyclic nucleus, was acquired from the culture medium of *Amanita virgineoides* (Asano et al. 2013). Three new bisabolanes **115**−**117** with dicyclic skeleton were isolated from edible mushroom *Pleurotus eryngii* (Kikuchi et al. 2018), whereas from another Chinese edible fungus *Pleurotus cystidiosus*, two new analogues featuring the octahydrobenzofuran moiety, pleuroton A (**118**) and pleuroton B (**119**), were obtained (Zheng et al. 2015). It seems that edible and medicinal fungi belonging to the genus *Pleurotus* is a potential producer of bisabolanes. Chromatographic separation of the fungal strain *A. tennesseensis* led to the isolation of three new bisabolane esters, aspertenols A−C (**120**−**122**) (Liu et al. 2018). **120** possessed a diphenyl ether moiety, while **121** and **122** contained a 3′,4′-dihydro-5-methoxysterigmatocystin substructure, representing the new type of phenolic bisabolane esters.

## Bisabolanes characterized from terrestrial plants

**Figure S2.** Bisabolanes characterized from terrestrial plants (**123**–**291**).

Phytochemical investigation on the rhizomes of *Curcuma longa* yielded a new phenolic bisabolane curcumalonone A (**123**) (Wu et al. 2020), while bisacurone D−G (**124**−**127**), four new bisabolanes were isolated and identified as two pairs of bisabolane stereoisomers from an extract of the rhizome of *C. longa* (Feng et al. 2020). From the stems and leaves of *Clausena sanki*, a new aromatic bisabolane, clausemargic A (**128**), was isolated and characterized (Hu et al. 2020). A new bisabolane named as 2α-acetoxy-1α,9α-oxidobisbol (**129**) with an oxo-bridge between C-1 and C-9 was isolated from the roots and rhizomes of *Valeriana amurensis* (Dong et al. 2021). A new β-bisabolol type monocyclic bisabolane, (1*R*,7*S*)-1,12,13-trihydroxybisabola-3,10-diene (**130**), was isolated from the stems and branches of *Viscum coloratum* (Chen et al. 2020). Cephasinene A (**131**), a new bicyclic bisabolane with an α,β-unsaturated γ-lactone ring, was obtained from the endemic species of *Cephalotaxus sinensis* (Ahmed et al. 2018). Eight new bisabolanes, namely pararunines C−J (**132**−**139**), were isolated from the whole plant of *Parasenecio rubescens* (Jin et al. 2017). **132**−**139** were highly oxygenated monocyclic bisabolanes bearing a cyclohexane ring. The isolation of these compounds may be of great chemotaxonomic significance of *P. rubescens*. Two new bisabolanes, (3*R*,4*S*,5*R*,7*S*)-3,4,5-trihydroxybisabola-10-ene-9-one (**140**) and (1*R*,2*S*,5*R*,7*R*,8*S*)-2,8-epoxy-5-hydroxybisabola-3,10-diene-9-one (**141**), were isolated from rhizomes of *C. longa* (Cheng et al. 2019). Three new monocyclic sesquiterpenes with a terminal C=C bond and an epoxy group, which were named as songaricalarins F−H (**142**−**144**), were isolated from roots and rhizomes of *Ligularia songarica* (Dai et al. 2015). A novel hydroxylated bicyclic bisabolane ketol, which was named as bicycloturmeronol (**145**), was isolated from turmeric *C. longa* (Del Prete et al. 2016). A new bisabolane bisabola-2,7(14),11-trien-10-ol (**146**) was obtained through the study of chemical composition of the essential oil from *Laggera pterodonta* (Compositae) (Gu et al. 2014). Three new bisabolanes, 6-(2-hydroxy-6-methylhept-5-en-2-yl)-3-(hydroxymethyl)-4-oxocyclohex-2-en-1-yl acetate (**147**) and 3-(hydroxymethyl)-6-(5-(2-hydroxypropan-2-yl)-2-methyltetrahydrofuran-2-yl)-4-oxocyclohex-2-en-1-yl acetate (**148** and **149**), were isolated from the leaves of American shrub *Lindera benzoin* (L.) Blume (Lauraceae) (Ryen et al. 2020). Compounds **148** and **149**, products of oxidization of **147** to form a tetrahydrofuran ring, were obtained as a diastereomeric mixture. Two new rare enone-containing bisabolanes, beshanzuenones C (**150**) and D (**151**), were isolated from the barks of the endangered plant *Abies beshanzuensis* (Hu et al. 2016). **150** and **151** possessed an unusual 6/6/5-fused tricyclic ring system. Two new bisabolanes, (+)-β-sesquiphellandrene-12-oic acid (**152**) and 4-oxo-lanceolic acid (**153**), together with a new norbisabolane, (+)-2-methyl-6[4-oxo-2-cyclohexen-1-yl]-2-(*E*)-heptenoic acid (**154**), were isolated from the leaves of *Ocotea minarum* (Nogueira et al. 2021). Two new phenolic bisabolanes, (6*S*)-2-hydroxy-6-(4-hydroxy-3-methylphenyl)-2-methylheptan-4-one (**155**) and (6*S*)-6-(4-hydroxy-3-methylphenyl)-2-methoxy-2-methylheptan-4-one (**156**), were obtained from the rhizomes of *C. longa* (Li et al. 2015), while four novel monocyclic bisabolanes, turmerones A−D (**157**−**160**), were also isolated from this species (Wen et al. 2018). Two new monocyclic bisabolanes, 6,13-dihydroxybisabola-2,10-diene (**161**) and 7,13-dihydroxybisabola-2,10-diene (**162**), were isolated from the heartwood of *Santalum austrocaledonicum* (Santalaceae) (Alpha et al. 1997)*.* Chemical studies on *Cremanthodium rhodocephalum* (Asteraceae) afforded three new bisabolanes, 10-hydroperoxybisabola-2,7(14),11-triene (**163**), 11-hydroperoxybisabola-2,7(14),9*E*-triene (**164**), and 8-acetoxy-3,4-epoxy-1-((*E*)-3-methylpent-2-enoyloxy)bisabola-7(14),10-dien-2-one (**165**) (Saito et al. 2012). **163** and **164** were the first cases of bisabolanes with hydroperoxides, while **165** was elucidated as bicyclic with an epoxide ring.

A pair of new norbisabolene enantiomers, (+)-curcumane D (**166**) and (−)-curcumane D (**167**), were isolated from *C. longa* (Qiao et al. 2019). **166** and **167** possessed a rare 15-norbisabolene skeleton with a chromone core. Two new bisabolanes, (7*R*,10*R*)-10,11-dihydro-10,11-dihydroxyxanthorrhizol 3-*O*-β-_D_-glucopyranoside (**168**) and (−)-curcuhydroquinone 2,5-di-*O*-β-_D_-glucopyranoside (**169**), were isolated from the rhizomes of *Curcuma xanthorrhiza* (Zingeberaceae) (Park et al. 2014). **168** was identified as a sesquiterpene monoglycoside with a β-glucopyranose, while **169** contained two glucopyranoses. Phytochemical investigation of the leaves of *Lychnophora ericoides* (Asteraceae) led to the isolation of a new *orto*-acetoxy-bisabolol (**170**) (Pavarini et al. 2013). A new highly oxygenated norbisabolane named phyllaemblinol (**171**) was isolated from the fruits of *Phyllanthus emblica* (Euphorbiaceae) (Zhang et al. 2016). A pair of new bisabolane (6*S*,7*S*)-3-hydroxy-3-hydroxymethylbisabola-1,10-diene-9-one (**172**) was isolated from *C. longa* (Ti et al. 2021). Five new bisabolanes, which were identified as (8β,10α)-8-(angeloyloxy)-5,10-epoxybisabola-1,3,5,7(14)-tetraene-2,4,11-triol (**173**), (8β,10α)-8-(angeloyloxy)-5,10-epoxythiazolo[5,4-*a*]bisabola-1,3,5,7(14)-tetraene-4,11-diol (**174**), (1α,2α,3β,5α,6β)-1,5,8-tris(angeloyloxy)-10,11-epoxy-2,3-dihydroxybisabol-7(14)-en-4-one (**175**), (1α,2α,3β,5α,6β)-2,5,8-tris(angeloyloxy)-10,11-epoxy-1,3-dihydroxybisabol-7(14)-en-4-one (**176**), and (1α,2β,3β,5α,6β)-1,8-bis(angeloyloxy)-2,3-epoxy-5,10-dihydroxy-11-methoxybisabol-7(14)-en-4-one (**177**), were isolated from the roots of *Ligularia dentata* (Compositae) (Baba et al. 2007). Differed from the monocyclic **175**−**177**, compound **173** was a dicyclic bisabolane comprising a benzene ring and a seven-numbered ring, whereas **174** contained an additional thiazole ring fused to the benzene ring, which represented the first sesquiterpenoid with the rare benzothiazole moiety. From the roots of *Cremanthodium campanulatum* (Asteraceae), two new bisabolanes, 1β,5α,8-trisangeloyloxy-3β,4β,10,11-bisepoxybisabol-7(14)-en-2-one (**178**) and 2β,8-bisangeloyloxy-3β,4β,10,11-bisepoxybisabol-7(14)-en-1β-ol (**179**), were isolated and characterized (Saito et al. 2019). Both compounds contained a highly oxygenated cyclohexane ring and two or three angeloyloxy moieties. An intensive phytochemical investigation on the traditional herb *Tussilago farfara* afforded five new bisabolanes **180**−**184** (Song et al. 2021). All of them contained angeloyloxy and/or isobutyryloxy groups. Moreover, **184** was the uncommon example of halogenated bisabolanes. Two new epoxide bisabolanes, 2β-acetoxy-1β,8-diangeloyloxy-3β,4β-epoxy-10,11-dihydroxybisabol-7(14)-ene (**185**) and 2β-acetoxy-1β,8-diangeloyloxy-3β,4β-epoxy-10-hydroxy-11-methoxybisabol-7(14)-ene (**186**), were isolated from *Cremanthodium discoideum* (Compositae) (Zhu et al. 2000). Phytochemistry study of the whole plants of *Ligularia thyrsoidea* (Compositae) yielded five new chlorinated bisabolanes, 1β-acetoxy-2β,8-diangeloyloxy-3β-hydroxy-4α-chloro-10,11-expoxybisabol-7(14)-ene (**187**), lβ-acetoxy-2β,10-diangeloyloxy-3β,8,11-trihydroxy-4α-chlorobisabol-7(14)-ene (**188**), 1β-acetoxy-2β,8-diangeloyloxy-3β,10-dihydroxy-4α-chloro-11-ethoxybisabo1-7(14)-ene (**189**), 1β-acetoxy-2β,8-diangeloy1oxy-3β,10-dihydroxy-4α-chloro-11-methoxybisabol-7(14)-ene (**190**), and 1β-acetoxy-2β,8-diangeloyloxy-3β,10,11-trihydroxy-4α-chlorobisabol-7(14)-ene (**191**) (Liao et al. 1999), whereas five analogues **192**−**196** were isolated from the same *Ligularia* species (*L. lankongensis*) (Onuki et al. 2008). These compounds were polysubstituted types with a monocyclic skeleton and a terminal double bond. Furthermore, five chlorine-bearing congeners **197**−**201** were isolated from *Cremanthodium discoideum* (Zhu et al. 1999). Systematical phytochemical studies on *Ligularia cymbulifera* led to the isolation and identification of 15 new highly oxygenated bisabolanes **202**−**216** (Wu et al. 2012; Liu et al. 2008; Liu et al. 2006). Finally, 22 new ones, pararubins A−V (**217**−**238**), were obtained from the whole plants of *Parasenecio rubescens* (Compositae) (Jin et al. 2015). This kind of compounds was firstly isolated from the genus *Parasenecio*.

Altaicalarins A−D (**239**−**242**), four new bisabolanes were isolated from the roots and rhizomes of *Ligularia altaica* (Asteraceae) (Wang et al. 2010). Four new polysubstituted bisabolanes, namely (1*R**,2*S**,4*R**,5*S**)-4-(acetyloxy)-2-[3-(acetyloxy)-1,5-dimethylhex-4-enyl]-5-methylcyclohexyl (2*Z*)-2-methylbut-2-enoate (**243**), (1*R**,4*S**,6*R**)-4-(acetyloxy)-6-[3-(acetyloxy)-1,5-dimethylhex-4-enyl]-3-methylcyclohex-2-en-1-yl (2*Z*)-2-methylbut-2-enoate (**244**), and 3-methyl-1-{2-[(1*R**,2*R**,5*R**,6*S**)-2,5,6-tris(acetyloxy)-4-methylcyclohex-3-en-1-yl]propyl}but-2-enyl (2*Z*)-2-methylbut-2-enoate (**245** and **246**), were isolated from the roots of *Leontopodium alpinum* (Asteraceae) (Stuppner et al. 2002). Three new bisabolanes with a hexenyl side chain, *rel*-(1*S*,4*R*,5*S*,6*R*)-4,5-diacetoxy-6-[(*R*)-1,5-dimethylhexa-3,5-dienyl]-3-methylcyclohex-2-enyl (*Z*)-2-methylbut-2-enoate (**247**), *rel*-(1*S*,4*R*,5*S*,6*R*)-4,5-diacetoxy-6-[(*R*)-5-hydroxy-1,5-dimethylhex-3-enyl]-3-methylcyclohex-2-enyl (*Z*)-2-methylbut-2-enoate (**248**), and *rel*-(1*R*,2*S*,4*R*,5*S*)-4-acetoxy-2-[(*R*)-5-hydroxy-1,5-dimethylhex-3-enyl]-5-methylcyclohexyl (*Z*)-2-methylbut-2-enoate (**249**), were isolated from the roots of *Leontopotium longifolium* (Compositae) (Li et al. 2006). Two new bisabolanes with an epoxy-ketone ring system, (3*R*,4*R*,6*S*)-3,4-epoxybisabola-7(14),10-dien-2-one (**250**) and (1*R*,3*R*,4*R*,5*S*,6*S*)-1-acetoxy-8-angeloyloxy-3,4-epoxy-5-hydroxybisabola-7(14),10-dien-2-one (**251**), were isolated from the flower buds of *Tussilago farfara* (Compositae) (Yaoita et al. 2001). Two new aromatic bisabolanes, (6*S*)-2-methyl-6-(4-hydroxyphenyl)-2-hepten-4-one (**252**) and (6*S*)-2-methyl-6-(4-formylphenyl)-2-hepten-4-one (**253**), were isolated from the rhizomes of *Curcuma longa* (Zeng et al. 2007). Two bisabolanes, hamanasal A (**254**) and hamanasol A (**255**), were isolated from leaves of *Rosa woodsii* (Hashidoko et al. 1992). Bisacurol B (**256**) was isolated from the rhizome of *Curcuma domestic*a (Ishii et al. 2011), while three new analogues, (1*R*,7*R*)-1,12-dihyroxybisabola-3,10-diene (**257**), (1*R*,7*S*)-1,12-dihyroxybisabola-3,10-diene (**258**), and 7,12,13-Trihydroxybisabola-3,10-diene (**259**), were obtained from the heartwood of *Santalum album* (Santalaceae) (Kim et al. 2005). A new natural bisabolane intermedin B (**260**) was isolated from the aerial parts of *Schisandra propinqua* (Li et al. 2008). From the rhizomes and roots of *Valeriana jauriei*, two new bisabolanes, (1*R*,2*R*,7*R*)-2-acetoxyl-β-bisabolol (**261**) and (1*R*,2*R*,7*R*)-2-hydroxyl-β-bisabolol (**262**) were obtained (Nishiya et al. 1994). Phytochemical survey of the aerial parts of *Lippia dulcis* (Verbenaceae) afforded eight new bisabolanes with a cyclohexenone framework, designated lippidulcine A (**263**) and epilippidulcine A (**264**) (Ono et al. 2005), peroxylippidulcines A−C (**265**−**267**), peroxyepilippidulcine B (**268**), and epilippidulcines B (**269**) and C (**270**) (Ono et al. 2006). A new chlorinated bisabolane (6*R*)-2-chloro-6-[(1*S*)-1,5-dimethylhex-4-en-1-yl]-3-methylcyclohex-2-en-1-one (**271**) was isolated from the roots of *Euphorbia chrysocoma* (Euphorbiaceae) (Shi et al. 2005). Osterivolones A−D (**272**−**275**), four novel bisabolanes were obtained through the bioactivity-guided isolation of the roots of *Angelica koreana* (Umbelliferae) (Lee et al. 2012). **272**, **273**, and **275** featured a dicyclic bisabolangelone skeleton, while **274** was an uncommon asymmetric dimer of bisabolanelone derivatives. A novel bisabolane, glochicoccin A (**276**), was isolated from the rhizomes of *Glochidion coccineum* (Euphorbiaceae) (Xiao et al. 2007). **276** was established as a highly oxygenated bisabolane with a 6/5/6/5 tetracyclic ring system. Another four new bisabolangelones, liginvolones A−D (**277**−**280**), were isolated from the roots of *Ligusticum involucratum* (Umbelliferae) (Shibano et al. 2005). **278** and **279** were regarded as a new type of bisabolane, in which an oxygen bridge was formed between C-3 and C-6. Three novel highly oxygenated bisabolanes possessing an aglycon, phyllaemblic acids B (**281**), C (**282**) and phyllaemblicin D (**283**), were isolated from the roots of *Phyllanthus emblica* (Euphorbiaceae) (Zhang et al. 2001). **283** was the 15-*O*-glucoside of **282**. Chemical study of the roots of *Phyllanthus emblica* (Euphorbiaceae) resulted in the isolation of eight novel highly oxygenated bisabolane glycoside phyllaemblicins G1−G8 (**284**−**291**) (Lv et al. 2014). **285** was a rare bisabolane glycoside, in which possessing a tricyclo[3.1.1.1] oxygen bridge framework, whereas **289**−**291** were novel dimeric glycosides bearing two norbisabolane subunits by a disaccharide.

## Bisabolanes characterized from others

**Figure S3.** Bisabolanes characterized from others (**292**–**296**).

Apart from above-mentioned producers, five bisabolanes (**292**–**296**) were also isolated from the liverwort (Figure S3). Analysis of the essential oil of the liverwort *Radula perrottetii* belonging to the Jungermanniales (Hepaticae) discovered four new monocyclic bisabolanes, bisabola-2,6,11-triene (**292**), bisabola-1,3,5,7(14),11-pentaene (**293**), bisabola-1,3,5,7,11-pentaene (**294**), and 6,7-epoxybisabola-2,11-diene (**295**) (Tesso et al. 2005). Finally, a new bisabolane julaceal (**296**) was isolated from the liverwort *Anthelia julucea* (Nagashima et al. 1993).

**References**

Alpha T, Raharivelomanana P, Bianchini JP et al (1997) Bisabolane sesquiterpenoids from *Santalum austrocaledonicum*. Phytoehemistry 44:1519−1522.

Ahmed A, Li W, Zhang JS et al (2018) A new bisabolane sesquiterpenoid and a new abietane diterpenoid from *Cephalotaxus sinensis*. Nat Prod Res 32:175−181.

Asano M, Yamada K, Tanaka T et al (2013) New bisabolane sesquiterpene from the mycelia of *Amanita virgineoides*. Chem Pharm Bull 61:366−369.

Baba H, Yaoita Y, Kikuchi M (2007) Sesquiterpenoids and lactone derivatives from *Ligularia dentata*. Helv Chim Acta 90:1028−1037.

Chen YY, Hou JP, Huang L et al (2020) Chemical constituents of *Viscum coloratum* (Kom.) Nakai and their cytotoxic activities. Nat Prod Res (Online ahead of print, doi: 10.1080/14786419.2020.1837816)

Chen HP, Dong WB, Feng T et al (2014) Four new sesquiterpenoids from fruiting bodies of the fungus Inonotus rickii. J Asian Nat Prod Res 16:581−586.

Cheng X, Li H, Wu P et al (2019) Two new bisabolane-type sesquiterpenoids from the cooking liquid of *Curcuma longa* rhizomes. Phytochem Lett 29:169−172.

Dai W, Xie ZJ, Li M et al (2015) Three new bisabolane sesquiterpenes from *Ligularia songarica*. Helv Chim Acta 98:410−416.

Del Prete D, Millán E, Pollastro F et al (2016) Turmeric sesquiterpenoids: Expeditious resolution, comparative bioactivity, and a new bicyclic turmeronoid. J Nat Prod 79:267−273.

Dong FW, Li F, Ren JJ et al (2021) Sesquiterpenoids from the roots and rhizomes of *Valeriana amurensis* and their effects on NGF-induced neurite outgrowth in PC12 cells. Nat Prod Res 35:757−762.

Erkel G, Becker U, Anke T et al (1996) Nidulal, a novel inducer of differentiation of human promyelocytic leukemia cells from *Nidula candida*. J Antibiot 49:1189−1195.

Gu JL, Li ZJ, Zhang HX et al (2014) Fragrant volatile sesquiterpenoids isolated from the essential oil of *Laggera pterodonta* by using olfactory-guided fractionation. Chem Biodivers 11:1398−1405.

Guo ZY, Tan MH, Liu CX et al (2018) Aspergoterpenins A–D: four new antimicrobial Bisabolane sesquiterpenoid derivatives from an endophytic fungus *Aspergillus versicolor*. Molecules 23:1291.

Feng LP, Lu LH, Yuan MR et al (2020) Two pairs of bisabolane sesquiterpenoid stereoisomers, bisacurone D−G, from the rhizome of *Curcuma longa* L. Fitoterapia 146:104701.

Kikuchi T, Kitaura K, Katsumoto A et al (2018) Three bisabolane-type sesquiterpenes from edible mushroom *Pleurotus eryngii*. Fitoterapia 129:108−113.

Lee JW, Yun CY, Roh E et al (2012) Melanogenesis inhibitory bisabolane-type sesquiterpenoids from the roots of *Angelica koreana*. Bioorg Med Chem Lett 22:2927−2931.

Li J, Wang HF, Chen G et al (2015) Structure determination of two new bisabolane-type sesquiterpenes from the rhizomes of *Curcuma longa* by NMR spectroscopy. Magn Reson Chem 53:536−538.

Li HM, Lei C, Luo YM et al (2008) Intermedins A and B; new metabolites from *Schisandra propinqua* var. *intermedia*. Arch Pharm Res 31:684−687.

Li JX, Lin CJ, Yang XP et al (2006) New bisabolane sesquiterpenes and coumarin from *Leontopodium longifolium*. Chem Biodivers 3:783−790.

Liao JC, Zhu QX, Yang H et al (1999) Bisabolane Sesquiterpenes from *Ligularia thyrsoidea*. J Chin Chem Soc 46:185−190.

Liu CM, Wang HX, Wei SL et al (2008) Pyrrolizidine alkaloids and bisabolane sesquiterpenes from the roots of *Ligularia cymbulifera*. Helv Chim Acta 91:308−316.

Liu CM, Fei DQ, Wu QH et al (2006) Bisabolane sesquiterpenes from the roots of *Ligularia cymbulifera*. J Nat Prod 69:695−699.

Liu L, Liu R, Basnet BB et al (2018) New phenolic bisabolane sesquiterpenoid derivatives with cytotoxicity from *Aspergillus tennesseensis*. J Antibiot 71:538−542.

Lv JJ, Wang YF, Zhang JM et al (2014) Anti-hepatitis B virus activities and absolute configurations of sesquiterpenoid glycosides from *Phyllanthus emblica*. Org Biomol Chem 12:8764.

Ishii T, Matsuura H, Kaya K et al (2011) A new bisabolane-type sesquiterpenoid from *Curcuma domestica*. Biochem Syst Ecol 39:864−867.

Jin A, Wu W, Ruan H (2017) Sesquiterpenoids and monoterpenoid coumarins from *Parasenecio rubescens*. RSC Adv 7:5167.

Jin A, Wu WM, Yu HY et al (2015) Bisabolane-type sesquiterpenoids from the whole plant of *Parasenecio rubescens*. J Nat Prod 78:2057−2066.

Kim TH, Ito H, Hatano T et al (2005) Bisabolane- and santalane-type sesquiterpenoids from *Santalum album* of Indian origin. J Nat Prod 68:1805−1808.

Hashidoko Y, Tahara S, Mizutani J (1992) Bisabolane sesquiterpenes and a 2-phenoxychromone from *Rosa Woodsii* leaves. Phytochemistry 31:2148−2149.

Hu S, Ma YL, Guo JM et al (2020) Bisabolane sesquiterpenes from *Clausena sanki* with their potential anti-inflammatory activities. Nat Prod Res 34:3499−3505.

Hu CL, Xiong J, Li JY et al (2016) Rare sesquiterpenoids from the shed trunk barks of the critically endangered plant *Abies beshanzuensis* and their bioactivities. Eur J Org Chem 2016:1832−1835.

Nagashima F, Sari Y, Tori M et al (1993) Sesquiterpenoids from some European liverworts. Phytochemistry 34:1341−1343.

Nishiya K, Kimura T, Takeya K et al (1994) Sesquiterpenoids and iridoid glycosides from *Valeriana fauriei*. Phytochemistry 36:1547−1548.

Ono M, Morinaga H, Masuoka C et al (2005) New bisabolane-type sesquiterpenes from the aerial parts of *Lippia dulcis*. Chem Pharm Bull 53:1175−1177.

Ono M, Tsuru T, Abe H et al (2006) Bisabolane-type sesquiterpenes from the aerial parts of *Lippia dulcis*. J Nat Prod 69:1417−1420.

Onuki H, Yamazaki M, Nakamura A et al (2008) Chemical constituents and diversity of *Ligularia lankongensis* in Yunnan Province of China. J Nat Prod 71:520−524.

Park JH, Jung YJ, Mohamed MAA et al (2014) New bisabolane sesquiterpenes from the rhizomes of *Curcuma xanthorrhiza* Roxb. and their inhibitory effects on UVB-induced MMP-1 expression in human keratinocytes. Helv Chim Acta 97:438−446.

Pavarini DP, Nogueira EF, Callejon DR et al (2013) Novel bisabolane derivative from “arnica-da-serra” (*Vernonieae*: Asteraceae) reduces pro-nociceptive cytokines levels in LPS-stimulated rat macrophages. J Ethnopharmacol 148:993−998.

Qiao MM, Liu F, Liu Y et al (2019) Curcumane C and (±)-curcumane D, an unusual *seco*-cadinane sesquiterpenoid and a pair of unusual *nor*-bisabolane enantiomers with significant vasorelaxant activity from *Curcuma longa*. Bioorg Chem 92:103275.

Qu HR, Yang WW, Zhang XQ et al (2020) Antibacterial bisabolane sesquiterpenoids and isocoumarin derivatives from the endophytic fungus *Phomopsis prunorum*. Phytochem Lett 37:1−4.

Ryen AH, Göls T, Steinmetz J et al (2020) Bisabolane sesquiterpenes from the leaves of *Lindera benzoin* reduce prostaglandin E2 formation in A549 cells. Phytochem Lett 38:6−11.

Saito Y, Takiguchi K, Gong X et al (2012) Three new bisabolane-type sesquiterpenoids from *Cremanthodium rhodocephalum* (Asteraceae). Heterocycles 86:497−503.

Saito Y, Ichihara M, Takiguchi K et al (2019) Bisabolane, oplopane, and lignan constituents of *Cremanthodium campanulatum* collected in China. Nat Prod Commun 14: 1−7.

Shi HM, Long BS, Cui XM et al (2005) A new bisabolane sesquiterpenoid from *Euphorbia chrysocoma*. J Asian Nat Prod Res 7:857−860.

Shibano M, Okuno A, Taniguchi M et al (2005) Bisabolane-type sesquiterpenes: liginvolones A−D from *Ligusticum involucratum*. J Nat Prod 68:1445−1449.

Song XQ, Yu JH, Sun J et al (2021) Bioactive sesquiterpenoids from the flower buds of *Tussilago farfara*. Bioorg Chem 107:104632.

Sun LT, Chen Y, Yang HX et al (2020) Bisabolane sesquiterpenes and α-pyrone derivative from endophytic fungus *Zopfiella* sp. Phytochem Lett 37:29−32.

Stuppner H, Ellmerer EP, Ongania KH et al (2002) Bisabolane derivatives from *Leontopodium alpinum*. Helv Chim Acta 85:2982−2989.

Stierle AA, Stierle DB, Kemp K (2004) Novel sesquiterpenoid matrix metalloproteinase-3 inhibitors from an acid mine waste extremophile. J Nat Prod 67:1392−1395.

Tesso H, König WA, Asakawa Y (2005) Composition of the essential oil of the liverwort *Radula perrottetii* of Japanese origin. Phytochemistry 66:941−949.

Ti H, Mai Z, Wang Z et al (2021) Bisabolane-type sesquiterpenoids from *Curcuma longa* L. exert anti-influenza and anti-inflammatory activities through NF-κB/MAPK and RIG-1/STAT1/2 signaling pathways. Food Funct 12:6697−6711.

Nogueira CR, Carbonezi LH, de Oliveira CTF et al (2021) Sesquiterpene derivatives from *Ocotea minarum* leaves. Phytochem Lett 42:8−14.

Wang Q, Chen TH, Bastow KF et al (2010) Altaicalarins A−D, cytotoxic bisabolane sesquiterpenes from *Ligularia altaica*. J Nat Prod 73:139−142.

Wen J, Qiu TY, Yan XJ et al (2018) Four novel bisabolane-type sesquiterpenes from *Curcuma longa*. J Asian Nat Prod Res 20:928−933.

Wu YX, Chen YJ, Liu CM et al (2012) Four new sesquiterpenoids from *Ligularia cymbulifera*. J Asian Nat Prod Res 14:1130−1136.

Wu SH, Zhao LX, Chen YW et al (2011) Sesquiterpenoids from the endophytic fungus *Trichoderma* sp. PR-35 of *Paeonia delavayi*. Chem Biodivers 8:1717−1723.

Xiao HT, Hao XY, Yang XW et al (2007) Bisabolane-type sesquiterpenoids from the rhizomes of *Glochidion coccineum*. Helv Chim Acta 90:164−170.

Yaoita Y, Suzuki N, Kikuchi M (2001) Structures of new sesquiterpenoids from Farfarae Flos. Chem Pharm Bull 49:645−648.

Yang J, Wang N, Yuan HS et al (2013) A new sesquiterpene from the medicinal fungus *Inonotus vaninii*. Chem Nat Compd 261−263.

Zeng Y, Qiu F, Takahashi K et al (2007) New sesquiterpenes and calebin derivatives from *Curcuma longa*. Chem Pharm Bull 55:940−943.

Zhao ZZ, He LQ, Chen HP et al (2016) A new bisabolane-type sesquiterpenoid from the fermentation broth of fungus *Antrodiella gypsea*. J Asian Nat Prod Res 18:184−188.

Zhang Y, Zhao L, Guo X et al (2016) Chemical constituents from *Phyllanthus emblica* and the cytoprotective effects on H_2_O_2_-induced PC12 cell injuries. Arch Pharm Res 39:1202−1211.

Zhang YJ, Tanaka T, Iwamoto Y et al (2001) Novel sesquiterpenoids from the roots of *Phyllanthus emblica*. J Nat Prod 64:870−873.

Zheng Y, Pang H, Wang J et al (2015) New apoptosis-inducing sesquiterpenoids from the mycelial culture of Chinese edible fungus *Pleurotus cystidiosus*. J Agric Food Chem 63:545−551.

Zhu Y, Yang L, Jia ZJ (1999) Novel highly oxygenated bisabolane sesquiterpenes from *Cremanthodium discoideum*. J Nat Prod 62:1479−1483.

Zhu Y, Zhu QX, Jia ZJ (2000) Epoxide sesquiterpenes and steroids from *Cremanthodium discoideum*. Aust J Chem 53:831−834.
